# Explainable AI Algorithms for Vibration Data-Based Fault Detection: Use Case-Adadpted Methods and Critical Evaluation

**DOI:** 10.3390/s22239037

**Published:** 2022-11-22

**Authors:** Oliver Mey, Deniz Neufeld

**Affiliations:** 1 Fraunhofer IIS/EAS, Fraunhofer Institute for Integrated Circuits, Division Engineering of Adaptive Systems, 01187 Dresden, Germany; 2 Cognitive Systems Group, University of Bamberg, 96050 Bamberg, Germany

**Keywords:** condition monitoring, explainable AI, fault detection, machine learning, order analysis, vibration analysis

## Abstract

Analyzing vibration data using deep neural networks is an effective way to detect damages in rotating machinery at an early stage. However, the black-box approach of these methods often does not provide a satisfactory solution because the cause of classifications is not comprehensible to humans. Therefore, this work investigates the application of the explainable AI (XAI) algorithms to convolutional neural networks for vibration-based condition monitoring. Thus, the three XAI algorithms GradCAM, LRP and LIME with a modified perturbation strategy are applied to classifications based on the Fourier transform as well as the order analysis of the vibration signal. The following visualization as frequency-RPM maps and order-RPM maps allows for an effective assessment of saliency values for variable periodicity of the data, which translates to a varying rotation speed of a real-world machine. To compare the explanatory power of the XAI methods, investigations are first carried out with a synthetic data set with known class-specific characteristics. Both a visual and a quantitative analysis of the resulting saliency maps are presented. Then, a real-world data set for vibration-based imbalance classification on an electric motor, which runs at a broad range of rotation speeds, is used. The results indicate that the investigated algorithms are each partially successful in providing sample-specific saliency maps which highlight class-specific features and omit features which are not relevant for classification.

## 1. Introduction

Rotating machinery finds application in many domains, from production facilities, wind turbines, automotive to aerospace and many more. Degradation and aging of machine components is a perpetual process. Occurrence of failures is only a matter of time and determined by the runtime and load of a machine. However, once a defect develops in one of its sub-components, not only the functionality of the system is at risk: The whole system will experience stronger vibrations and increased vibrational load, causing in turn an increased degradation rate of other components. Therefore, it is important to detect and eliminate defects as early as possible [1,2,3].

Faults in rotating machinery can lead to periodically occurring signatures in the vibration data of a system, which enables non-invasive and real-time condition monitoring of the state of a machine [4,5,6,7]. In this work, vibration data are transformed to frequency-RPM and order-RPM maps [8,9], that can be used to classify states and defects using machine learning (ML) and artificial intelligence (AI) [10,11]. In principle, this is already possible using hand-designed features and threshold values [12,13]. However, manual feature extraction and design requires a lot of effort and expert knowledge of the inner workings of the machine [14].

Deep learning algorithms on the other hand have the advantage that they require less effort in terms of data preprocessing and expert knowledge of the specific machine [15,16,17,18,19,20]. However, deep learning necessitates a large amount of diverse training data for each defect type. The collection of training data with defect information is often challenging and expensive. In addition, the vibration behavior of a machine can differ significantly depending on fabrication parameters, a.o. screw tightening torque [21]. This can be remedied by disassembling and reassembling the system between recordings of data. If no such changes are conducted, the variability of the collected vibration data is low and measuring for longer amounts of time will not provide more relevant information. Models are therefore prone to overfitting and have a high chance of providing inaccurate classifications when applied to data from another machine of the same type. Understanding the decision process of a ML model is therefore crucial to identify cases where predictions are based on incorrectly learned input-output relationships. This is especially relevant for deep neural networks, used in deep learning.

The classification process of large deep learning models is usually not comprehensible to humans. Overfitting of a model or classifications based on wrong reasons can stay hidden until the model is applied to new data. Explainable AI (XAI) algorithms aim to overcome this by making the classification process transparent [22,23,24]. Many XAI algorithms were initially developed for application in image recognition [25]. Examples are GradCAM (Gradient-weighted Class Activation Mapping) [26], LRP (layer-wise Relevance Propagation) [27], LIME [28], DeepLift [29], Integrated Gradients [30], or SHAP (Shapley Additive Explanations) [31]. Applied in image recognition tasks, the goal of XAI is to highlight areas of input images, which were particularly important in the model’s decision for a certain class. These visualizations are also called saliency maps. To apply these algorithms to condition monitoring, they have to be transferred to the case of one-dimensional data, i.e., time series. Still, it needs to be considered that saliency methods in some cases produce inconsistent results, as reported by Kindermans et al. [32]. Since machine diagnosis can be used in safety-critical areas, it is important that the AI and ML algorithms used are verified as well as possible.

Artificial intelligence and deep learning is a relevant topic in the domain of rotating machinery diagnosis [33]. First demonstrations of the transfer of XAI techniques to machine fault diagnosis have already been reported. Chen et al., used GradCAM to explain fault classifications of a convolutional neural network, which are based on short time Fourier transformed vibration data [34]. GradCAM was further applied to explain bearing fault classifications based on preprocessed acoustic emission data [35], vibration data based classifications of a neuro-fuzzy network [36] and in [37] GradCAM was applied in an anomaly detection use-case for time series of vibration data of a rotating system. GradCAM was also applied to explain vibration data-based fault detection of linear motion guides [38], ball bearings [39] and grinding machines [40]. Further, frequency activation maps were calculated to explain a time domain-based bearing fault detection model in frequency domain [41]. LRP was used to explain fault classifications based on vibration data from a gearbox [42] and of multi-sensor information from a motor [43]. SHAP was used in [44] to explain bearing fault classifications of a k-nearest neighbor classifier. LIME has been used in spectral analysis on a deep neural network (DNN) with tabular, pre-selected features [45]. Furthermore, LIME was applied on the STFT map of vibration data [46] in order to highlight important pixels in the data, which is different to our approach that focusses LIME for frequency and order bands in the spectra. Attention mechanisms were utilized to explain bearing fault classifications from time domain vibrational data in [47,48].

For conventional image classification tasks, the evaluation of such an analysis is easily possible for humans as the ground truth is just the area of the object to be classified. However, the comparison with a ground truth for the case of vibrational data is not obvious. A comparison of different XAI algorithms for measurement data is therefore challenging. This motivates the introduction of a data set for vibration data-based fault detection, where not only the fault states are known but also the corresponding features or frequencies that are relevant to the classification of each class.

**Figure 1:**
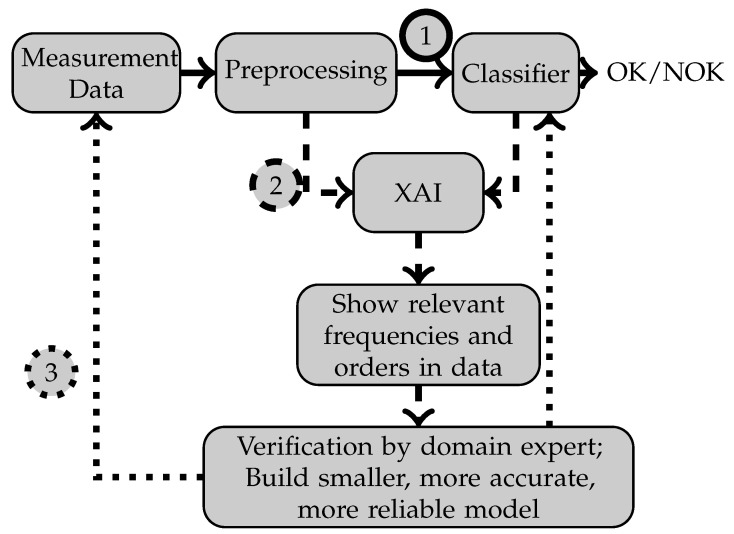
After the training in step (**1**), in step (**2**) XAI methods are used to generate saliency maps for the explanation of features. In step (**3**) the explanation is shown to domain experts, and their judgement is used to reduce the number of input features for future models.

As an application scenario, our work could be used for condition monitoring systems on rotary machines, in which the frequencies determining the condition classifications are also visualized. A process visualization for such a condition monitoring system is shown in Figure 1. Since for many defects such as bearing damage or imbalances signals occur at known points within the order spectrum, a plant engineer can understand whether the determined condition classification is plausible or not. Mistakes made by the classification model can thus be detected directly, whereupon either the model is improved or human expertise is further relied on for certain situations. From a developer’s perspective, the additional information from the XAI algorithms can also be used to restrict the input of machine learning models to specific frequency ranges. Frequencies, which are not relevant to any classification seem to not provide any useful information to the classification model and can thus be omitted.

This work aims to evaluate the plausibility of XAI methods explaining deep neural networks applied to vibration-based condition monitoring with a special focus on machines with variable rotation speed. The contributions of this paper are as follows:

- We created a synthetic data set based on sinusoidal data, allowing for an intuitive comparison of XAI algorithms applied to a supervised classification task based on this data set to a ground truth. Visualized as frequency-RPM map as well as an order-RPM map, rotation speed-dependent and -independent modes can be separated visually.
- We investigated the XAI algorithms GradCAM, LRP, and modified version of LIME in a comparative manner based on a visual and quantitative evaluation using the mentioned synthetic data set and a real-world imbalance detection data set.
- As a side result, the highest classification accuracy on the imbalance data set reported so far was improved to 99.66%.

To increase reproducibility, the source code of all analyses is published at Github [49]. In the Appendix A to this paper, the studies shown are supplemented with results from additional XAI methods.

## 2. Methods

In the following, the applied transformations of the data into frequency-RPM and order-RPM representations are described. Subsequently, the convolutional neural networks-based classifier models and the applied XAI approaches are explained.

### 2.1. Data Transformations into Frequency-RPM and Order-RPM Representations

Changes in a rotating system—like defects in its ball bearings or an evolving imbalance—cause distinct changes in the Fourier spectrum of its vibrational signal [9]. Here, we conducted both a transformation into a frequency-space representation as well as into an order space representation to be utilized as classification input.

For the frequency space representation, a frequency-RPM transformation was conducted. It is intended for signals from systems with increasing rotational speed, and is defined as the repeated application of the Fast Fourier Transform (FFT) to a small temporal subsection of the signal, similar to a Short-time Fourier transform (STFT). This yields a better temporal resolution compared to the application of the FFT to the complete signal at once. With rising RPM, resonance frequencies characteristic to the system appear in the corresponding frequency-RPM map as lines parallel to the time axis. Conversely, lines in the map that belong to resonance frequencies that stem from rotating components such as motors, pumps or ball bearings, are sloped curves with frequencies linear to the rising RPM of the system [9].

The order-RPM transformation is similar to the frequency-RPM transformation, with the difference that here the transformed signal is interpolated to a representation normalized to the current rotation speed of the system. Visualized into an order-RPM map, the *x*-axis of this plot would be given in orders with an order of 1 corresponding to the current RPM value. This way, the correlation of spectrum lines inverses: Lines parallel to the time axis belong to rotating subcomponents. Oscillations of constant frequency appear as curved lines [8,9]. The explained transformations were performed in Matlab using the rpmfreqmap and rpmordermap methods.

The resolution of the frequency-RPM maps was chosen such that the resulting map has a satisfactory temporal and frequential resolution to the human eye when visualized. To make the classification results comparable, the resolution of the order-RPM representation was chosen such that the resulting feature vectors have roughly as many elements as the frequency-RPM representation.

### 2.2. Classification Algorithms

**Figure 2:**
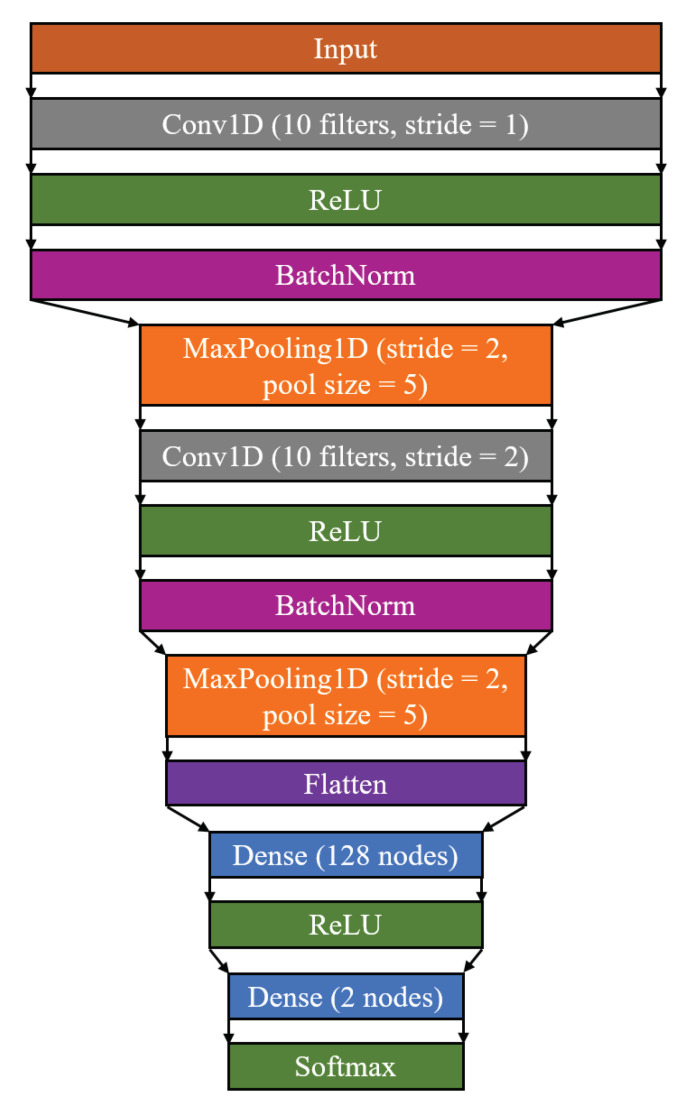
Layer-by-layer neural network architecture of the used classification models. While the general architecture is identical for the considered classification tasks, the dimension of each layer differs due to the task-specific dimension of the input vector.

The classification of the data sets was performed using CNNs. This allows the application not only of model-agnostic XAI methods such as LIME but also DNN-specific ones such as GradCAM and LRP. The network architecture used is shown in Figure 2. The models consist of two convolutional blocks, each with a convolutional layer and a ReLU activation function followed by batch normalization and a MaxPooling layer. Then, a flattening layer is applied and, after a dense layers with ReLU activation, the final classification is provided using a dense layer with two output units with a softmax activation function. The models were each trained for 150 epochs using the Adam optimizer with a learning rate of $10^{-4}$. A model checkpoint was used to store the model weights with the best prediction accuracy obtained during the training process on the model selection data subset.

### 2.3. XAI Algorithms

The XAI algorithms used in this work are GradCAM, LRP-Z and LIME. Additionally, the Appendix A contains similar investigations for GradCAM++, ScoreCAM, LRP-ε and LIME with other perturbation strategies.

Class Activation Maps (CAM) [50] are extracted by calculating the weighted sum of the feature maps of the last convolutional layer. However, for CAM it is required that a global average pooling layer and the final output layer directly follow the last convolutional layer. This obstacle was overcome by the introduction of GradCAM [26], where the gradients between the last convolutional layer and the output nodes are used to weight the individual filter channels for the calculation of the overall activation map. Since GradCAM relies on the gradients to weight the feature maps, no activation map can be extracted for cases, where no gradient exist, i.e., super-confident predictions. To remedy those, label smoothing was applied to the cross-entropy loss used for model optimization. This means that the target values for a specific class are not 0 or 1 anymore but in our case 0.05 and 0.95. This makes it possible to also extract a gradient from the final softmax layer in case of numerically required finite precision.

LRP (layer-wise relevance propagation) by Bach et al. [27] is a method aimed at nonlinear networks, such as convolutional neural networks. Based on a classifier and an input image, it provides a relevance score per pixel. Backtracking from the output layer of the network, this method computes the influence on the result based on the prior layer, layer for layer, until the input of the network. This way, the importance of each input pixel is determined. The basic version of LRP is called LRP-Z. There are several modifications of LRP such as LRP-ϵ, while LRP-Z based on the implementation of Reference [51] was selected due to the clearer saliency maps, as was determined in our experiments (not further described in this paper).

Local Interpretable Model-Agnostic Explanations (LIME) by Ribeiro et al. [28] is a model-agnostic method for quantifying feature importance. It perturbs the input data instance several times in random places by replacing parts of it with, e.g., the data set’s average, noise, or zeros. After applying the trained model to the new data again, a ridge regression model is used to find the perturbations that influence the model’s outcome the most. This way, the important parts of the original instance can be found. LIME has been applied in related work to tabular, text, time series and image data.

**Figure 3:**
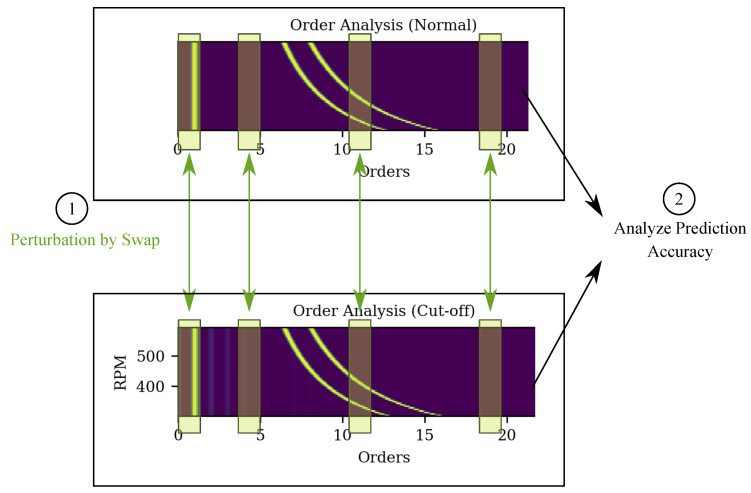
Schematic on the proposed perturbation strategy. For the input data of each class, data in the segment is replaced by data from the opposite class at the same segment position in the spectrum.

Our goal to visualize important frequencies and orders in the spectrum. Therefore, our modified version of LIME presented here is the first to focus on the analysis of bands in the frequency and order maps. This is is motivated by the fact that certain types of defects in a system cause vibrations, which result in lines of the spectral maps that are parallel to the RPM axis. The implementation from LIME for time series was used as a base [52] and adaptions as described below were introduced. The implementation in [52] focuses on the analysis of one time series at a time, and splits it into a preset amount of segments, which are then perturbed. This approach would be specific for one sample of the dataset, and therefore computationally expensive for large data sets. Due to our large amount of data, this would not be effective. To remedy this, the fact that certain frequency and order bands are important for the anomaly classification of rotating systems can be leveraged. Therefore, to overcome the limitation due to large data, instead we propose a strategy for LIME (Global LIME) for global feature importance for RPM-maps. Here, LIME is applied to the complete input data set of one class at once. In the frequency domain, this means that certain frequency bands are perturbed, and the same goes analogously for the order analysis. To generate examples of higher fidelity, instead of, e.g., noise or zeros, the data in the bands is perturbed by replacing it with the data from the opposite class’ data at the same position (as shown in Figure 3). Other replacement strategies (noise, zeros, average) are investigated in the Appendix A.

In the original Lime-for-time implementation [52], the amount of segments *n* and the amount of features *f* act as hyperparameters. In our experiments, it became apparent that the choice of these parameters had a large impact on the analysis results, making the method less robust. To achieve more consistent results than with just one configuration, we do not just choose one combination of *n* and one *f*. Instead, we generate LIME evaluations for multiple segment counts $n_{i}$ and feature counts $m_{j}$. This way, i∗j intermediate output saliency maps are created. The final saliency map is computed by averaging all resulting maps using their mean. This is conducted for all LIME applications in this paper (including for other perturbation strategies in Appendix A).

### 2.4. Visualization

The goal of XAI is to provide humans with insights into how black-box machine learning algorithms work. A crucial aspect in the use of XAI methods is therefore the processing of the additional information obtained. In this work, the determined saliency values are visually processed in frequency-RPM maps and order-RPM maps. Using the Python library Matplotlib, all diagrams were created as heatmaps with the Viridis [53] color map. Its advantages are a perceptually uniform representation of colors and a good translation to grayscale, so it is suitable even in case of color blindness.

Prior to plotting, the saliency values obtained as output from the applied XAI methods were preprocessed to maximize the visibility of the contained patterns. All XAI output data was first normalized to a range between 0 and 1. For GradCAM and LRP-Z, only positive values were used to mitigate visual noise while this was not necessary for LIME. All results were visualized using linear scaling, while applying minimum and maximum value cropping based on quantiles to optimize the visiblity of the saliency maps.

## 3. Results

A synthetic data set, which allows for evaluations of XAI algorithms with known ground truth, as well as a data set obtained from measurements at a rotating shaft were used for the presented investigations. Both data sets and the corresponding strategies for partitioning into subsets for training, model selection, and testing are discussed in Section 3.1. The classification models as introduced in Section 2.2 were trained subsequently as described in Section 2. In the following, the testing accuracies achieved in each case are calculated and the outputs of different XAI algorithms, which are supposed to explain the classifications of the models, are evaluated.

### 3.1. Data Sets

Two different data sets are demonstrated. One is based on a superposition of sinusoidal functions and is designed as part of this work to serve as an evaluation tool of the investigated XAI methods. The second one is an actual vibrational data set recorded from a rotating machine.

#### 3.1.1. Sine Cut-Off Classification

**Figure 4:**
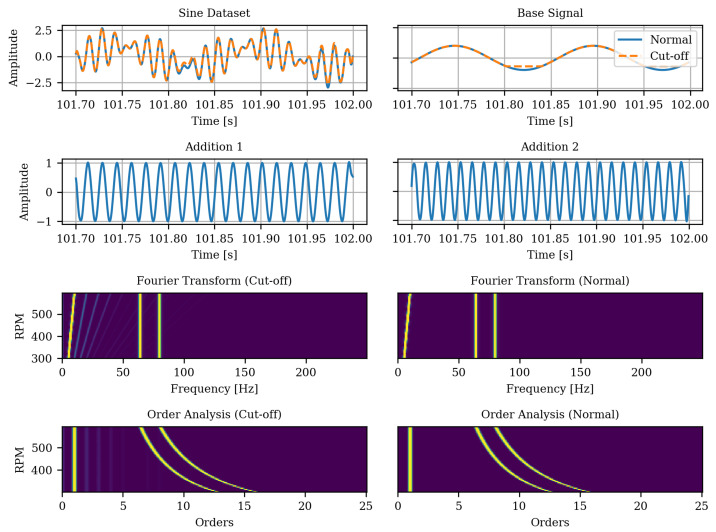
Sine data set components. Either the “Normal” or “Cut-Off” data are used and “Addition 1” and “Addition 2” are added.

**Figure 5:**
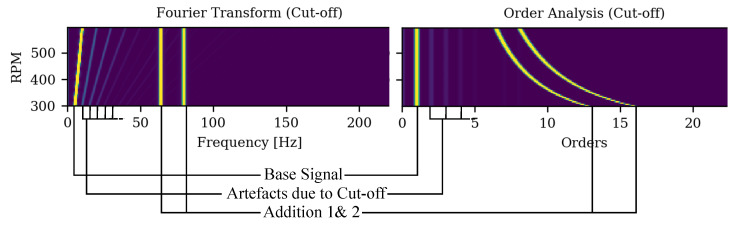
Elements within the spectrum of the Sine cut-off dataset in the frequency-RPM map and the order-RPM map representation.

In typical XAI and saliency map applications, the output for the classified image is intuitively verifiable and usually corresponds to the object in the image or characteristic features of it. Vibration data are only understandable given domain knowledge of the underlying system and signal processing. However, a visual comparison of different XAI algorithms is difficult without intuitive input data. We therefore introduce a synthetic data set for a binary classification of a periodic time series. It consists of additively combined sinusoidal functions, as shown in Figure 4. The main component is a chirp (sine with linearly increasing frequency), which is truncated at values of amplitude <−0.7 to 0.7 for one class which is referred to as the *cut-off* class. The class without truncation is referred to as the *normal* class. The two other components are two sine functions which have two different and constant, higher frequencies. They are added to both the normal and the cut-off sequences. In the time domain, which is however not considered in this paper, the truncated regions are the distinguishing feature and should be highlighted by an XAI algorithm. In the frequency domain, and thus in the order domain as well, the cut-off causes additional lines in the spectra at higher orders of the fundamental mode, as depicted in Figure 5). Accordingly, these should be highlighted by an XAI algorithm as distinguishing features. A total of 20% of the data was held back for testing and the remaining 80% were used for model development and split into 80% for model training and 20% for model selection during training.

#### 3.1.2. Imbalance Classification

The data set used for the demonstration of the methodology in a real-world application was introduced in [18]. It contains measurements obtained from vibration sensors attached to a drive train of a rotating motor, where imbalances of various strength were mounted. There are two measurements for each imbalance strength, one for model training and one for testing, respectively. During each measurement, the rotation speed of the drive train was gradually increased from a lower motor RPM to a higher RPM for two times. It thereby allows for a rotation-speed dependent evaluation of the classification accuracy of imbalance detection models. The data set is especially suited for this study due of its broad recorded rotation speed range. Further, its pre-defined split into training and test data allows for comparability between results of different studies. The data set is available at [54]. As with the sine cut-off data set, the training data was further divided into subsets of 80% used for model training and 20% used for model selection during the training process.

### 3.2. Classification Accuracy

The prediction accuracy of the models described in Section 2 were evaluated after their training using the respective test data sets. Results are shown in Table 1 and Table 2. In the case of the sine Cut-off data set, 100% classification accuracy was achieved both when using the Fourier transformed data and when using the order analysis. Apparently, the detection of the inserted disturbance in the sinusoidal signal is an easy task for CNNs. Test accuracies of 99.66% and 98.49% were achieved for the classification of the imbalance data set, using Fourier transform and order analysis, respectively. Thus, despite identical model architecture, higher classification accuracy was achieved with the Fourier transformed data. Still, both classification accuracies are higher compared to the best value achieved so far, which was reported to be 98.2% [18].

### 3.3. XAI Evaluation: Sine Cut-Off Classification

**Figure 6:**
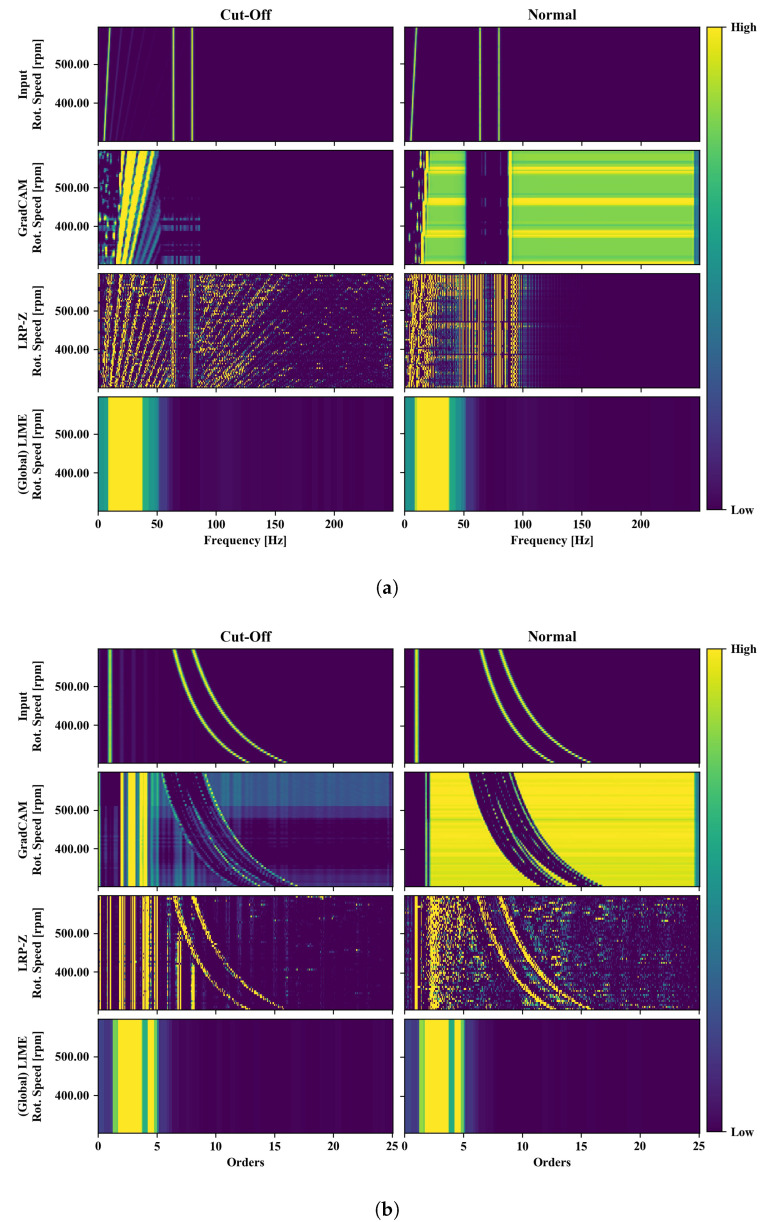
Visualization of the input data used for model training as well as saliency maps of three XAI methods applied to sine cut-off classification in a frequency-RPM-map (**a**) and an order-RPM-map (**b**).

Various post-hoc XAI methods are applied to the trained classification models. Figure 6 shows the raw frequency-RPM maps of the input values as well as the outputs of the XAI methods GradCAM, LRP-Z and (Global) LIME. The left column of the figure shows the data for the case with cut-off, the second column the data for the normal case (without cut-off).

For the case of the visualization, the signals corresponding to the basic sine function with increasing frequency, in the following called fundamental mode, are seen as the line in spectrum closest to the RPM-axis. The signals of the added sine functions with constant frequency (the two lines the farthest away from the RPM-axis, hereafter referred to as the superimposed modes) are also clearly visible.

While the *Cut-off* class is detectable at the higher orders of the fundamental mode, the *normal* class is detectable by the absence of those higher orders as well as by a higher amplitude of the fundamental mode. Ideally, an XAI algorithm would ignore the superimposed modes as they provide no distinguishing feature between the two classes.

The output of GradCAM fulfills the condition, that it should ignore the superimposed modes. Additionally, the higher orders of the fundamental mode are highlighted for the *cut-off* class. For the case of the class *normal*, areas are highlighted which seem to contain no class-specific information, especially for the case of frequencies higher than those of the superimposed modes.

LRP-Z highlights even more higher orders of the fundamental mode when classifying the *cut-off* class, while on the other hand also highlights the superimposed modes. Those superimposed modes, together with the fundamental mode, are also highlighted when classifying the *normal* class. This provides a better contrast than the original presentation of the input, but no additional information on the functioning of the CNN, since all pixel of high intensity are highlighted in the explanation.

The implementation of LIME for time series introduced in this paper provides global information about the relevance of individual features. Accordingly, in this case for the class *normal* the lowest regions of the spectrum should be highlighted, which also contain the fundamental mode. For the class *cut-off*, on the other hand, the areas between the fundamental mode and the superimposed modes should be highlighted. For both classes, the Global LIME variant highlights the regions of the spectrum that lie directly above the fundamental mode, i.e., the regions where the distortion caused by the cut-off occurs.

The same procedure was also applied to the order-RPM map of the data (shown in Figure 6b). The position of the fundamental mode is fixed to order 1 regardless of the rotational speed and therefore forms a vertical line in the diagram in the top row of Figure 6b. The disturbance caused by the cut-off of the sinusoidal function can be seen at integer numbers of the corresponding order higher than 1. The superimposed modes, on the other hand, form a curved line in the order-RPM map.

The output of GradCAM is similar to its application to the frequency-RPM map: For the *cut-off* class, the higher orders of the fundamental mode are highlighted and the superimposed modes are ignored. The unexpected highlighting of the higher order regions for the *normal* class also appears similarly, leaving out the irrelevant lines from the constant sine frequencies.

When LRP-Z is applied, both the fundamental mode and its higher orders and the superimposed modes are highlighted similar to the result in Figure 6a.

With Global LIME, the order range above the fundamental mode is highlighted for both classes, while the fundamental mode itself and the superimposed modes are not highlighted. This means that Global LIME correctly distinguishes between relevant and non-relevant features. However, due to the global nature of this explanatory method, a comparatively broad band is highlighted, since the entire frequency range within the sweep of the rotation speed needs to be covered.

In summary, for the synthetic data set considered here, Global LIME highlighted the relevant differences between the *normal* class and the *cut-off* class, but in a lower resolution than GradCAM and LRP-Z and without producing sample-specific explanations. GradCAM appeared to actually highlight important parts of the spectrum, but also highlighted non-relevant parts within the spectrum of the *normal* class. LRP-Z visualized the input values with a modified intensity and thereby made features of a smaller amplitude more visible, but failed to mark the superimposed modes as non-relevant.

Since this data set was synthetic, it is also possible to quantitatively validate the saliency maps. For this, the following terms are defined:

- **Spectral modes**: Spectral positions with top 80% of intensity.
- **Relevant pixels**: The absolute value of the subtraction of both spectra is calculated. The pixels with the top 80% values of the resulting map are referred to as **relevant pixels**.
- **Irrelevant pixels** are pixels with values >0.1 in the data from the *normal class* which are not at the same time **relevant pixels**.
- **Highlighted pixels** are pixels in the heatmaps with top 80% intensity.

**Figure 7:**
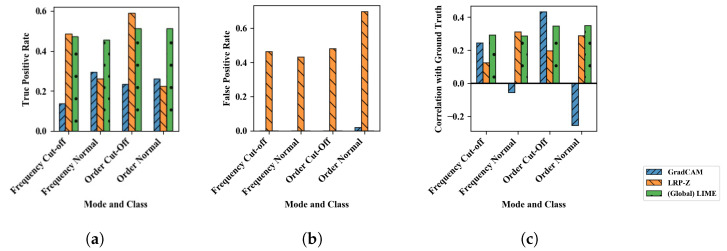
Evaluation of results for sine cut-off data set. (**a**) True-positives: Ratio of pixels correctly identified as relevant. (**b**) False-positives: Ratio of pixels incorrectly highlighted as relevant. (**c**) Correlation between the highlighted and the relevant pixels.

Given this, multiple metrics were evaluated as shown in Figure 7. Figure 7a shows for the applied XAI methods the true positive rate, i.e., the number of highlighted pixels which are also relevant pixels divided by the number of relevant pixels. Global LIME and LRP-Z score higher for this compared to GradCAM, as could be expected from the saliency maps shown before. In contrast, Figure 7b shows the false positive rate, i.e., the number of highlighted pixels which are also irrelevant pixels divided by the number of irrelevant pixels. Since LRP-Z generally highlighted a large part of the spectral modes, it obtains high values here, while GradCAM and Global LIME have almost no false positive pixels. Figure 7c shows the Pearson correlation between the heatmap values and the values of relevant pixels in the input. It is striking that GradCAM has a negative score for the normal classes for this metric. This probably stems from the fact that it highlights large parts of the spectrum at higher frequencies/orders.

### 3.4. XAI Evaluation: Imbalance Classification

**Figure 8:**
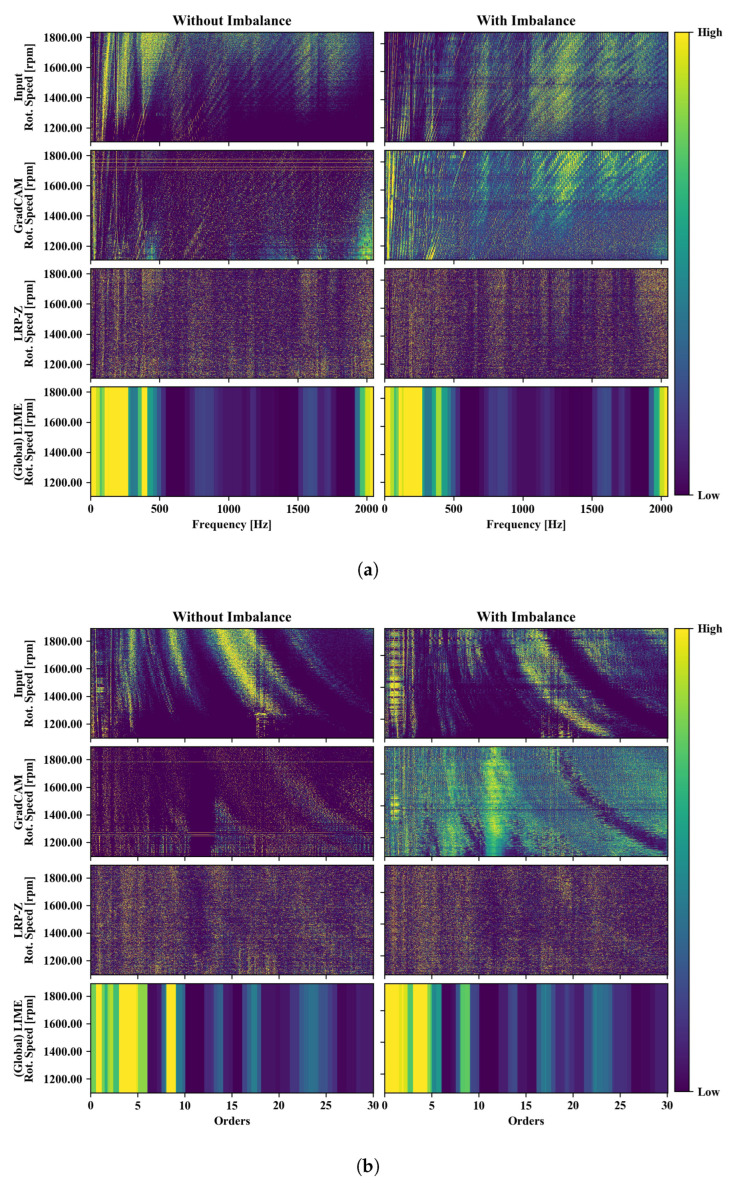
Visualization of the input data used for model training as well as saliency maps of three XAI methods applied to imbalance classification in a frequency-RPM-map (**a**) and an order-RPM-map (**b**).

The same XAI evaluation previously discussed for the sine cut-off classification was also performed for the imbalance classification. The corresponding heatmaps highlighting the input parts relevant for the classification according to different XAI algorithms are shown in Figure 8.

The FFT-transformed vibration values are displayed in Figure 8a. Both, diagonal lines corresponding to vibrations with a frequency proportional to the rotational speed of the drive train as well as lines with a constant frequency corresponding to resonances inside the setup are clearly visible for the cases with and without imbalance.

The application of GradCAM leads to a strong highlighting of the lower speed-dependent modes for the case of the imbalance class. This seems reasonable since an imbalance leads to oscillations with the periodicity of the rotational speed. For the imbalance free case a weak highlighting of the fundamental mode is present but not for its higher orders. The saliency map produced by GradCAM for the imbalance-free case on the other hand only highlights a small area with constant frequency at the upper edge of the frequency range. For the imbalance case, many higher-order rotation speed-dependent modes are highlighted indicating that those modes are also excited by the imbalance. Low emphasis is given to the modes with constant frequency. This behavior is similar to the observations made based on the application of GradCAM to the sine cut-off data set.

The response from LRP-Z, on the other hand, mainly shows modes with constant frequency. For both the classes with and without imbalance, almost no frequency dependence can be seen. Only for the case with imbalance, a weak emphasis on rotation speed-dependent modes is visible. Further, the difference in the visual appearance of the LRP-Z results of both classes is much weaker compared to GradCAM which makes it harder to interpret the classifier based on the LRP-Z result.

(Global) LIME highlights the frequency range between the fundamental mode, the lower range of the frequency spectrum (below 500 Hz). Similar to GradCAM, high importance is given to the highest obtained frequencies around 2000 Hz as well. Some less important bands can be seen at around 800 Hz, 1200 Hz and 1600 Hz.

The order analysis of the vibration data contains a range up to the 25th order, as depicted in Figure 8b. In the resulting diagrams, in particular the modes of constant frequency stand out as curved lines, while the modes with a frequency proportional to the rotation speed can be seen here as vertical lines. In the case of the imbalance class, an increased intensity of the lower speed-dependent modes can be seen clearly, analogous to the frequency representation.

In this case, compared with the Fourier transformed data, GradCAM highlights the rotation-speed dependent modes significantly less, making the explanatory saliency less informative. The contrast between relevant and non-relevant input parts according to GradCAM appears low while some focus is given on rotation speed-dependent modes. While the GradCAM visualization seems to be less informative compared to the frequency representation, LRP-Z is able to clearly highlight the first and the second mode for the imbalance class. This is a significant improvement in comparison to its output applied to the Fourier transformed data. However, it would not be reasonable to use the LRP-Z result for dimensionality reduction, since it appears that no part of the input is declared non-relevant. For this purpose, the Global LIME result depicted in the last row of Figure 8b seems more suitable, as there is a clear distinction between relevant and non-relevant parts of the data. Most relevant orders are under 10, but there are important bands at orders 14, 17 and around 24. Still, investigations would need to be carried out for this application to find out whether classification accuracy can still be maintained at a high level when removing all the input parts with zero feature importance.

## 4. Discussion

In general, frequency and order maps are already a means to provide additional insights into vibration signals, especially for the case of a system with variable rotational speed. By applying XAI methods on vibration data-based fault classification, further information can be gained about the fault-specific relevance of vibrational modes in different spectral positions. Still, an important factor for the interpretability of these algorithms is the scaling of their output, which makes them dependent on additional parameters such as thresholds and quantiles.

Due to the design of the sine cut-off data set, it was possible to assess the plausibility of various XAI algorithms applied to periodic time series. Given the conditions that saliency maps should only highlight class-specific features, omit non-distinguishing features and provide sample-specific explanations, each investigated XAI algorithm was only partially successful. It is also worth mentioning that the results from GradCAM and LRP-Z were complementary, which motivates the investigation of potential combinations of both methods.

The utilization of an artificially created data set, such as the sine cut-off data set, for the visual and computational evaluation of XAI algorithms can be a crucial tool to develop XAI algorithms specialized to be applied with data on periodic time series, or with relevant information in the frequency domain. Due to its design, the insights gained from the application of XAI methods on the sine cut-off data set can be transferred to other fault detection tasks encompassing periodic time series such as acoustic emission or motor voltages. On the other hand, the study using the sine cut-off data set showed that the information extracted should be taken with caution as some methods are able to correctly filter out non-relevant parts but also highlight features which do not contain information relevant for classification.

The investigations based on the imbalance data set demonstrated that the saliency values determined by means of XAI can shift in the frequency and order spectrum in proportion to the change in rotational speed. Still, a relatively huge share of the input data is marked as relevant for classification by the employed algorithms despite the fact, that imbalance is usually visible by a huge signal at the rotation frequency of the system. Future work could focus on reducing this proportion by maintaining high saliency values only for those features that cannot be removed without decreasing classification accuracy. Shapley values [31] as well as explanations based on attention mechanisms could help to achieve this goal.

For all evaluations of the saliency maps, it should be noted that only the behavior of the classification model is described. In the case of the Sine Cut-Off dataset, it was assumed that the trained classification models can perfectly represent the classification task due to its simplicity, thus allowing conclusions to be drawn about the plausibility of the XAI algorithms. The validity of the saliency maps tends to decrease the worse the classification model can represent the classification task. For the case of a practical application of the used XAI algorithms, the advantage for GradCAM and LRP is that a sample-wise inference with sufficient inference speed is achieved. In contrast, the LIME variant presented performs a calculation over the entire data set, which would be impractical in the case of a condition monitoring system with near real-time inference. Furthermore, the question of the applicability of comparable XAI methods for anomaly detection use cases, which are often a precursor to condition monitoring systems with integrated damage classification, remains open.

## 5. Conclusive Remarks

In many application areas, the black-box nature of deep neural networks motivates the development of methods that make machine learning-based classifications explainable. In the field of machine fault diagnosis, XAI algorithms promise to make deep learning models more comprehensible and thus more robust and powerful. Following this motivation, we investigated the application of the popular XAI methods GradCAM, LRP and LIME on the classification of vibration data transformed by means of an FFT as well as by an order analysis. To evaluate the plausibility of XAI results especially for the case of machines with variable rotation speeds, we designed and introduced the synthetic *sine cut-off data set* and further applied the given algorithms to a real-world imbalance detection dataset.

From the investigations conducted, we are able to draw the following conclusions:

- All considered XAI methods were partially able provide class-specific saliency maps which extract the class-distinguishing features while omitting those without class-specific information.
- Due to known class-specific information in the spectra, the synthetic *sine cut-off data set* allows for a quantitative and qualitative comparison of the characteristics of XAI algorithms applied to 1D-periodic data such as vibration-based condition monitoring with variable rotation speed.
- Frequency-RPM and order-RPM maps are an effective means to visually separate rotation speed-dependent modes and constant frequency system resonances.

Our study motivates the further development of explainable machine learning methods applied to condition monitoring use-cases, while also putting emphasis on verifying these algorithms with more understandable data sets as a proof of concept first.

## Figures and Tables

**Table 1:** Classification loss on test set. Bold numbers highlight the best result for each data set.

Data Set	Domain	
	Frequency	Order
Sine Cut-Off	**1.06**	5.26
Imbalance	**0.93**	1.23

**Table 2:** Classification accuracy on the test set. Bold numbers highlight the best result for each data set.

Data Set	Domain	
	Frequency	Order
Sine Cut-Off	100%	100%
Imbalance	**99.66** %	98.49%

**Figure A1:**
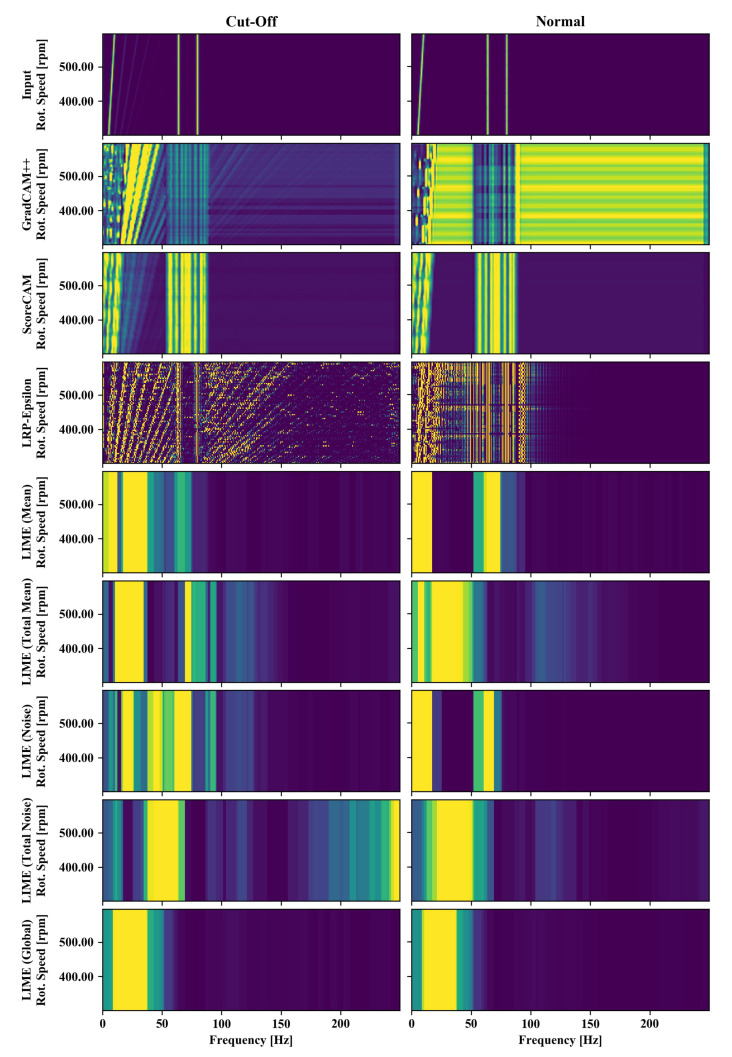
Visualization of saliency maps of the extended set of XAI methods applied to the sine cut-off classification in frequency representation.

**Figure A2:**
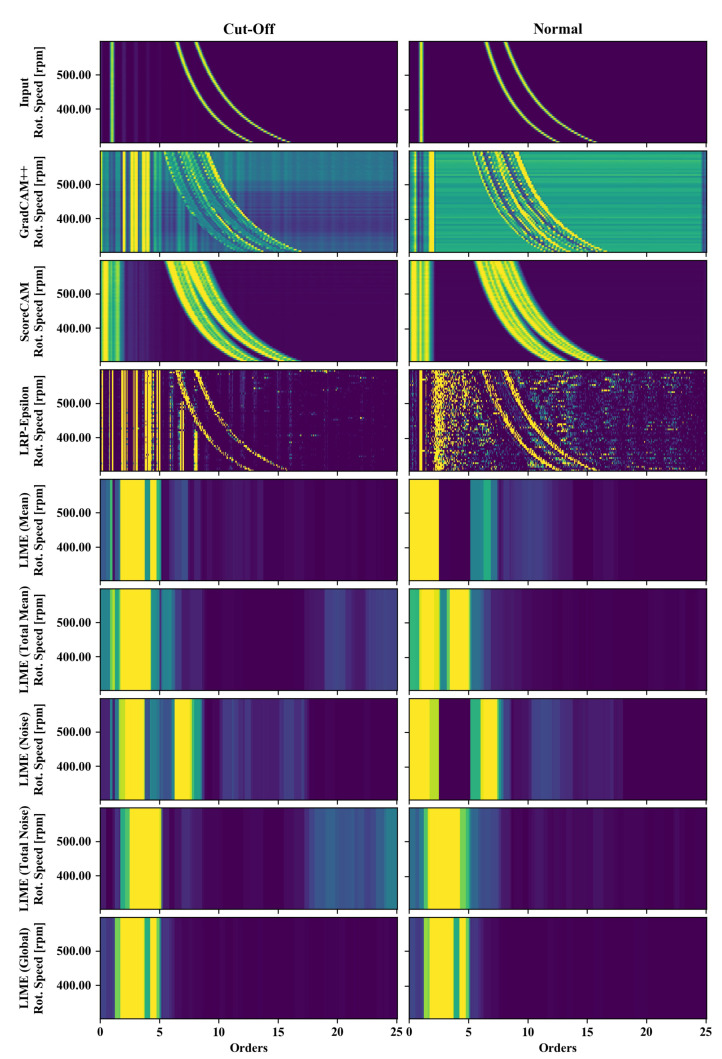
Visualization of saliency maps of the extended set of XAI methods applied to the sine cut-off classification in order representation.

**Figure A3:**
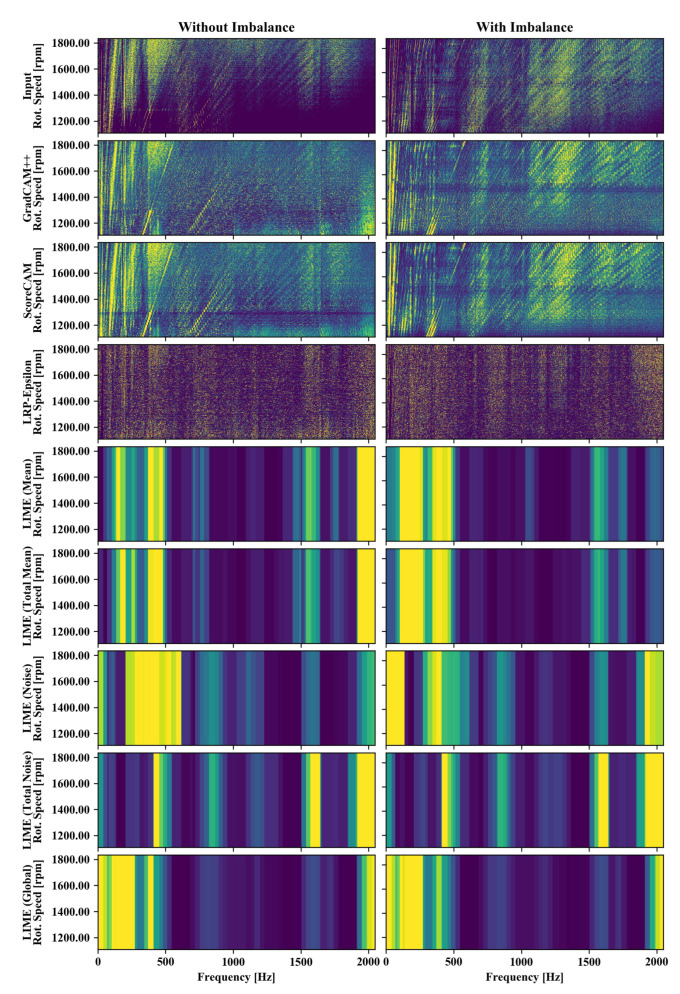
Visualization of saliency maps of the extended set of XAI methods applied to the imbalance classification in frequency representation.

**Figure A4:**
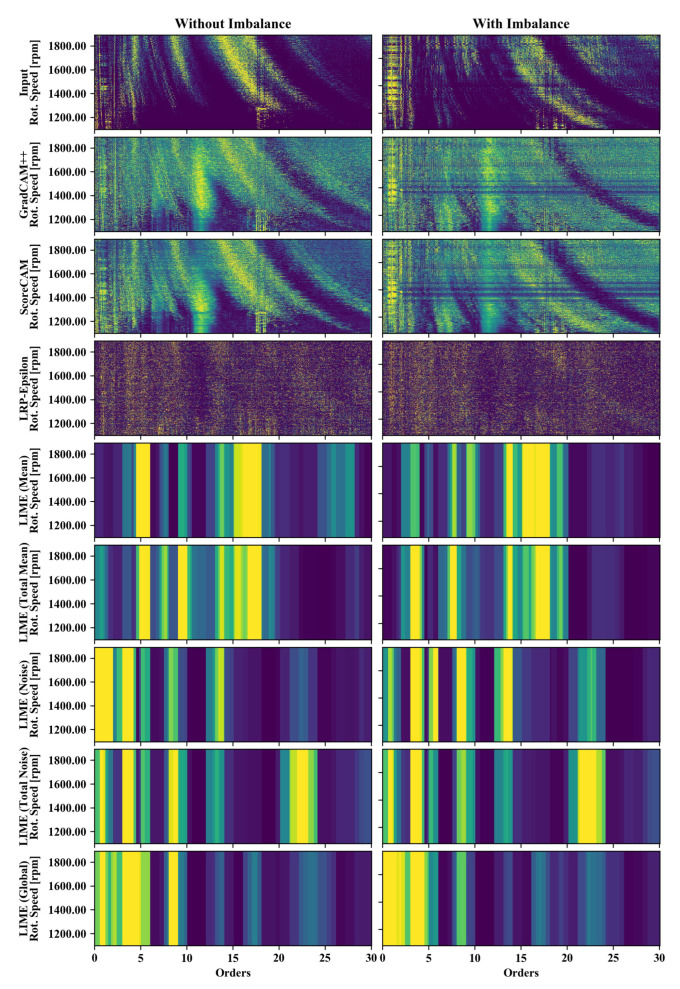
Visualization of saliency maps of the extended set of XAI methods applied to the imbalance classification in order representation.

## Data Availability

The source code to create the synthetic dataset described in this work is documented at [49]. The vibration dataset for unbalance classification can be downloaded from [54].

## Appendix A

In the following, the comparison of the different XAI algorithms is put on a broader basis. In addition to the methods GradCAM, LRP-Z, and the modified LIME implementation described in Section 2.3, the methods GradCAM++, ScoreCAM, and LRP-Epsilon were also applied and examined. GradCAM++ [55] modifies the calculation of the gradient-based weights to achieve better results for cases, where multiple instances of a certain class appear in a single data sample. ScoreCAM [56] overcomes the super-confidency issue by obtaining feature map weights by calculating the channel-wise increase of confidence of the model, when the original input is multiplied with the upsampled feature maps. ScoreCAM does therefore not depend on the model gradients. Further the authors showed that it localizes class-discriminant input areas more accurate compared to GradCAM and GradCAM++.

In addition, LIME was used similarly to the method described in the main section by replacing certain frequency bands and order bands; but instead of using values from the opposite class, noise with the same mean and standard deviation of the original data set was used.

The results of ScoreCAM and GradCAM++ were scaled in the same way as GradCAM from this paper, and LRP-Epsilon the same way as LRP-Z. The corresponding plots for the sine cut-off classification as well as for the imbalance classification for both the frequency and order representation are provided in the Figure A1, Figure A2, Figure A3 and Figure A4, respectively.

There are several different perturbation strategies reported for LIME. To give an impression of the performance of our method, some other standard perturbation strategies for LIME (in this case from [52]) are shown. The data are perturbed either with an average value or with uniform noise. The evaluated perturbation strategies are defined as follows:

- Mean: Mean value of the data from the original class at the perturbed sections
- Total Mean: Mean value of the complete data of the original class
- Noise: Noise with the value range from the data of the original class at the perturbed sections
- Total Noise: Noise with the value range from the complete data of the original class

It can be seen that the Global LIME method presented consistently highlights the important features in the data, particularly compared to other LIME perturbation strategies.
